# Quantification of Liver Fibrosis, Steatosis, and Viscosity Using Multiparametric Ultrasound in Patients with Non-Alcoholic Liver Disease: A “Real-Life” Cohort Study

**DOI:** 10.3390/diagnostics11050783

**Published:** 2021-04-26

**Authors:** Alexandru Popa, Felix Bende, Roxana Șirli, Alina Popescu, Victor Bâldea, Raluca Lupușoru, Radu Cotrău, Renata Fofiu, Camelia Foncea, Ioan Sporea

**Affiliations:** Department of Gastroenterology and Hepatology, ‘‘Victor Babeș’’ University of Medicine and Pharmacy, Piața Eftimie Murgu 2, 300041 Timișoara, Romania; popa.alexandru@umft.ro (A.P.); roxanasirli@gmail.com (R.Ș.); alinamircea.popescu@gmail.com (A.P.); victorbaldea07@gmail.com (V.B.); raluca_lupusoru@yahoo.ro (R.L.); cotrau.radu@yahoo.com (R.C.); renata.fofiu@yahoo.com (R.F.); foncea.camelia@gmail.com (C.F.); isporea@umft.ro (I.S.)

**Keywords:** liver fibrosis, liver steatosis, liver inflammation, multiparametric ultrasound, ultrasound-based elastography, viscosity, attenuation, sound speed

## Abstract

Non-alcoholic fatty liver disease (NAFLD) is the most common chronic liver disease worldwide. This study aimed to evaluate the performance of four ultrasound-based techniques for the non-invasive multiparametric (MPUS) assessment of liver fibrosis (LF), steatosis (HS), and inflammation in patients with NAFLD. We included 215 consecutive adult patients with NAFLD (mean age: 54.9 ± 11.7; 54.5% were male), in whom LF, HS, and viscosity were evaluated in the same session using four new ultrasound-based techniques embedded on the Aixplorer MACH 30 system: ShearWave Elastography (2D-SWE.PLUS), Sound Speed Plane-wave UltraSound (SSp.PLUS), Attenuation Plane-wave UltraSound (Att.PLUS), and Viscosity Plane-wave UltraSound (Vi.PLUS). Transient Elastography (TE) with Controlled Attenuation Parameter (CAP) (FibroScan) were considered as control. All elastographic measurements were performed according to guidelines. Valid liver stiffness measurements (LSM) were obtained in 98.6% of patients by TE, in 95.8% of patients by 2D-SWE.PLUS/Vi.PLUS, and in 98.1% of patients by Att.PLUS/SSp.PLUS, respectively. Therefore, 204 subjects were included in the final analysis. A strong correlation between LSMs by 2D-SWE.PLUS and TE (r = 0.89) was found. The best 2D-SWE.PLUS cut-off value for the presence of significant fibrosis (F ≥ 2) was 7 kPa. Regarding steatosis, SSp.PLUS correlated better than Att.PLUS with CAP values: (r = −0.74) vs. (r = 0.45). The best SSp.PLUS cut-off value for predicting the presence of significant steatosis was 1524 m/s. The multivariate regression analysis showed that Vi.PLUS values were associated with BMI and LSM by 2D-SWE.PLUS. In conclusion, MPUS was useful for assessing fibrosis, steatosis, and inflammation in a single examination in patients with NAFLD.

## 1. Introduction

Non-alcoholic fatty liver disease (NAFLD) was defined as a pathological entity in 1980. It was described as an excessive fat infiltration of the liver in the absence of significant alcohol consumption or other causes of liver disease. NAFLD is the most common chronic liver disease worldwide with an estimated global prevalence of 25%. Thus, it became a significant health and economic burden of modern society [1].

Being closely associated with a cluster of comorbidities such as central obesity, hypertension, type 2 diabetes, and dyslipidemia, NAFLD shares common pathophysiologic mechanisms with the metabolic syndrome (MetS). However, NAFLD is currently not a component of the diagnostic criteria for MetS. Thus, recently, the term “metabolic-associated fatty liver disease” (MAFLD) was suggested as a more appropriate substitute for NAFLD [2,3].

NAFLD ranges from simple steatosis to progressive steatohepatitis, which can advance to cirrhosis with consequent complications such as hepatocellular carcinoma [4]. Liver biopsy is still considered the gold standard method to discriminate simple steatosis from progressive steatohepatitis with fibrosis, but it is an invasive procedure with some drawbacks: need of expert practitioners, sampling errors, inter and intra-operator variability, high costs, and risk of complications. Due to these facts, it is not always accepted by patients, especially in the monitoring of disease progression [5,6]. As liver fibrosis in NAFLD is the most significant predictor of mortality, non-invasive, repeatable and precise methods for assessing steatosis (HS), fibrosis (LF), and inflammation can be of great clinical value [1].

Ultrasound-based liver elastography methods were implemented during the last 20 years and became well-accepted non-invasive LF assessment tools [7,8]. Transient Elastography (TE) (FibroScan, EchoSens, Paris, France) is the first and most validated technique developed, followed by other techniques, such as point Share Wave Elastography (pSWE), Two-Dimensional Share Wave Elastography (2D-SWE), or Time-Harmonic Elastography embedded in ultrasound systems [7,9,10,11,12,13,14].

2D-SWE allows a real-time qualitative and quantitative tissue elasticity evaluation by measuring the velocity of shear waves produced by a focused ultrasound beam. Supersonic Imagine (SSI) developed the first 2D-SWE method and numerous studies, and meta-analyses demonstrated its value for assessing LF [15,16,17,18,19,20].

The first diagnosis task in NAFLD is to assess the presence of HS. Magnetic resonance imaging-derived proton density fat fraction (MRI-PDFF) has significant diagnostic value for HS in patients with NAFLD and proved useful in the grading of HS with high sensitivity and specificity [21]. Quantitative multiparametric magnetic resonance imaging is an effective alternative to liver biopsy for diagnosing NASH and NAFLD, and it may offer clinical utility in patient management [22]. Thus, compared with ultrasound-based techniques, MRI has some well-known drawbacks such as higher costs, lower accessibility, more time-consuming and requires technical expertise to perform and interpret the readings. B-mode ultrasound is the most commonly used imaging tool for the evaluation of liver disease. It also proved to be a good semi-quantitative tool in the initial assessment of steatosis. The Controlled Attenuation Parameter (CAP) embedded in the FibroScan device was the first non-invasive quantitative tool developed for HS assessment. CAP analyzes the ultrasound attenuation through the liver. A good correlation between CAP and histologic steatosis grades in adults was demonstrated in numerous studies and meta-analyses [23,24,25]. Thus, it was recommended by the World Federation for Ultrasound and Medicine and Biology (WFUMB) as a standardized and reproducible point-of-care method for the detection of HS [7,9,10,11,26,27,28].

Ultrasound-based technologies that can quantify fibrosis and steatosis with the same device were developed by different manufacturers (General Electric, Hitachi, Canon) in the last couple of years [29,30,31].

SSp.PLUS is a new ultrasound-based method that allows HS assessment by estimating the intrahepatic speed of sound throughout a fixed region of interest (ROI). Att.PLUS mode displays information about tissue ultrasound beam attenuation through an ROI by measuring the decrease in the amplitude of ultrasound waves as they propagate throughout the tissue, as a function of frequency. Both techniques are embedded in the new Aixplorer MACH 30 system and are designed to be used for the non-invasive HS assessment.

Inflammation plays an essential role in the development of LF [32]. Vi.PLUS allows the display of information about tissue shear wave dispersion, which can serve as an indirect method for measuring viscosity [33].

Recently, it has been demonstrated that MPUS examinations have the potential to provide a comprehensive estimation of the main components of early-stage NAFLD [34].

This study aimed to evaluate the feasibility and the performance of four new ultrasound-based techniques (ShearWave Elastography, Sound Speed Plane-wave UltraSound, Attenuation Plane-wave UltraSound, and Viscosity Plane-wave UltraSound) embedded on the new Aixplorer MACH^®^ 30 system (Aixplorer, Supersonic Imagine, Aix-en-Provence, France), for the non-invasive assessment of LF, HS, and inflammation, using Transient Elastography (TE) with Controlled Attenuation Parameter (CAP) (FibroScan, EchoSens, Paris, France) as reference method.

## 2. Materials and Methods

### 2.1. Study Population

A monocentric cross-sectional study was performed during a 4-month interval (October 2020 to February 2021) in a tertiary Department of Gastroenterology and Hepatology. Based on the inclusion/exclusion criteria, 215 consecutive NAFLD patients (mean age: 54.9 ± 11.7; 54.5% male) were enrolled. Ultrasound-based measurements were performed in all patients, in the same session, using ShearWave Elastography (2D-SWE.PLUS), Sound Speed Plane-wave Ultrasound (SSp.PLUS), Attenuation Plane-wave Ultrasound (Att.PLUS), Viscosity Plane-wave Ultrasound (Vi.PLUS) from Aixplorer (Supersonic Imagine, Aix-en-Provence, France), and using TE with CAP (FibroScan, EchoSens, Paris, France). The study was approved by the Research Ethics Committee and the Institutional Review Board of our University (49/21.09–14.10.2020) and was performed following the World Medical Association Declaration of Helsinki, revised in 2000, Edinburgh. All the patients provided written informed consent before study entry.

The diagnosis of NAFLD and the inclusion/exclusion criteria were based on the American Association for the Study of Liver Diseases latest guidelines [9]. The inclusion criteria were age older than 18 years, fatty changes of the liver observed by abdominal ultrasound, agreement to participate in the study, and signed informed consent. Exclusion criteria were lack of informed consent, patients younger than 18, increased alcohol consumption (ethanol intake > 210 g per week for men and > 140 g per week for women), another chronic liver disease with clear etiology (hepatitis C virus infection, hepatitis B virus infection, autoimmune hepatitis, primary biliary cholangitis or primary sclerosing cholangitis), presence of ascites, biliary obstruction, elevated aminotransferase levels more than 5 times the upper normal limit, oncologic history or patients with focal liver lesions, and heart failure generating liver congestion.

Data gathered from the patients’ medical records included age, gender, BMI, abdominal circumference, complete blood counts, international normalized ratio (INR), thrombocytes, total bilirubin concentrations, aminotransferases levels, gamma-glutamyltransferase (GGT), albumin, cholesterol (HDLc, LDLc), and triglycerides.

With a minimum of 2 years of experience in elastography, two different physicians performed all measurements in the same session, blinded to each other’s results and the patients’ medical information. Liver stiffness, steatosis, and viscosity were assessed using 2 distinctive systems. ShearWave Elastography (2D-SWE.PLUS), Sound Speed Plane-wave Ultrasound (SSp.PLUS), Attenuation Plane-wave Ultrasound (Att.PLUS), and Viscosity Plane-wave Ultrasound (Vi.PLUS) were performed using the new Aixplorer MACH 30 system (SuperSonic Imagine, Aix-en-Provence, France), while TE and Controlled Attenuation Parameter (CAP) measurements were performed with the FibroScan system (EchoSens, Paris, France).

### 2.2. ShearWave PLUS Elastography

**Figure 1:**
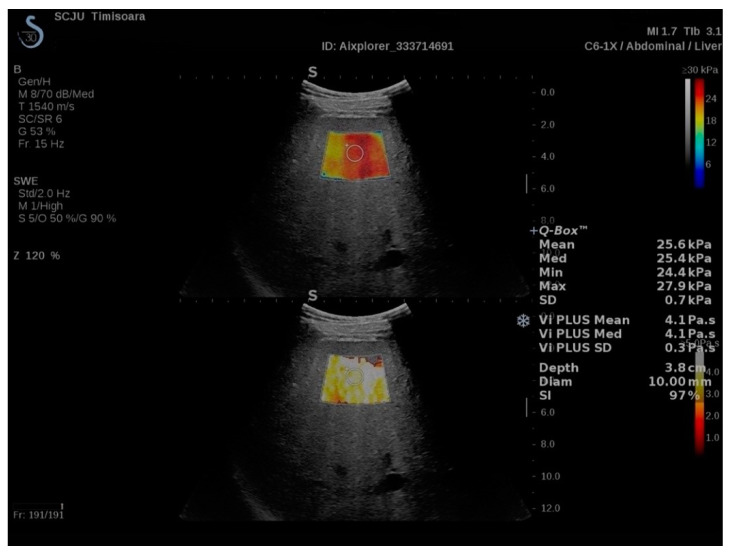
Illustration of a ShearWave PLUS Elastography (2D-SWE.PLUS) and Vi.PLUS measurement performed in a cirrhotic patient. Two color scale maps are displayed. In the SWE map, displayed in the upper part of the image, no fibrosis is color-coded with blue, while red signifies severe fibrosis. The viscosity is displayed in the lower part of the image. Colors close to yellow-white indicate high viscosity, while red signifies low viscosity, numeric 2D-SWE.PLUS results (expressed in kPa) and of Vi.PLUS (expressed in Pa.S) are displayed on the right side of the image. The Q-Box displays the mean, median, minimum, maximum, and standard deviation (SD) of the measurements, along with the depth, the diameter of the ROI, and the Stability Index (SI).

2D-SWE.PLUS measurements were performed with a C6-1X convex transducer using using the UltraFast™ software available on the Aixplorer Mach 30 ultrasound system, (Supersonic Imagine, Aix-en-Provence, France). According to the WFUMB and EFSUMB guidelines, all patients were examined in fasting conditions, in the supine position, with the right arm elevated above the head, by an intercostal approach, in the right liver lobe [6,7,30,35]. After selecting the best acoustic window by ultrasound examination and obtaining a suitable image, the shear-wave measurement box was positioned at least 1.5 cm below the right hepatic lobe capsule, avoiding large vessels or bile ducts and rib shadows. Acquisitions were performed during neutral respiratory apnea. After the SWE map was appropriate, image acquisition was performed, and then, the Q-Box was positioned in an area of relative uniform elasticity, at a depth of 3–5 cm (Figure 1). SSI developed a relatively new quality parameter, the measurement stability index tool (SI), derived from the spatial and temporal stiffness stability within the circular Q-Box. When the SI is lower than 90%, the manufacturer recommends placing the Q-Box elsewhere in the ROI or performing a new SWE acquisition. Thus, each SWE measurement was acquired at a stability index >90%. The median value of 5 SWE measurements (obtained from 5 different frames), expressed in kiloPascals (kPa), was considered as indicative of LS. An IQR to the median ratio (IQR/M) <30% was used as a measurement reliability criterion [8].

### 2.3. Viscosity PLUS

Vi.PLUS allows displaying information about tissue shear wave dispersion (analysis of shear wave propagation velocity at several frequencies). The extent of change in shear wave speed between frequencies is qualitatively represented in an easy to interpret the color-coded image and also quantitatively expressed in Pa.s over a range of values. As Vi.PLUS is combined with the SWE mode (Figure 1), acquisitions were made simultaneously with the SWE measurements and using the same protocol.

### 2.4. Attenuation PLUS and Sound Speed PLUS Break to Next Page

The Sound Speed Plane-Wave Ultrasound (SSp.PLUS) is a new technology that allows quantification of the intrahepatic speed of sound, reflecting fat content, which is an essential indicator for the detection and diagnosis of hepatic steatosis. Local measurement of the sound speed is expressed in m/s over a range of values (from 1450 to 1600 m/s).

The Attenuation Plane-Wave Ultrasound (Att.PLUS) mode displays information about tissue ultrasound beam attenuation through an ROI by measuring the decrease in amplitude of the ultrasound waves as they propagate through the tissue, as a function of frequency. The ultrasound beam attenuation information is quantitative. Local measurement of tissue attenuation is expressed in dB/cm/MHz over a range of values (from 0.2 to 1.6 dB/cm/MHz).

**Figure 2:**
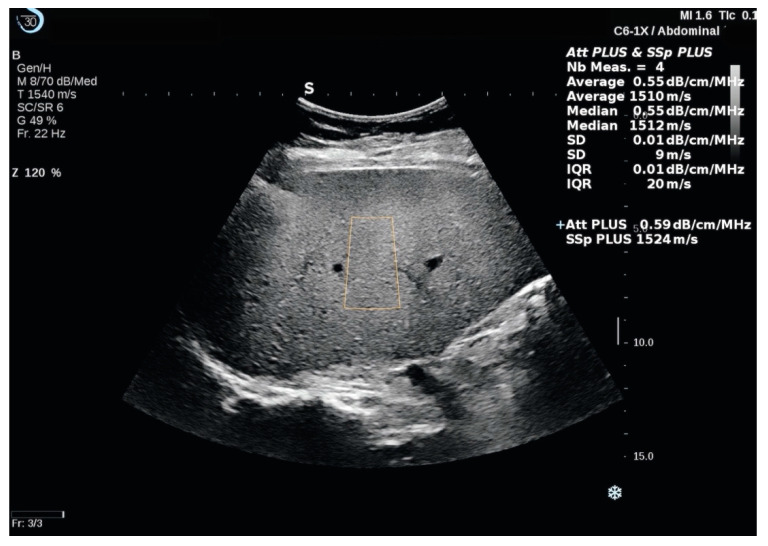
The Sound Speed Plane-Wave Ultrasound (SSp.PLUS) and the Attenuation Plane-Wave Ultrasound (Att.PLUS) measurements performed in a patient with liver steatosis using the Aixplorer MACH 30 platform. The Region of Interest (ROI) is positioned at a fixed depth, in an area free of major vessels or other structures. A measurement window with statistical values (Mean, Median, SD, and IQR) is displayed on the right side of the main screen. The results are expressed in m/s for SSp.PLUS and in db/cm/MHz for Att.PLUS.

Att.PLUS and SSp.PLUS are available in B-mode on a live image. Both of the modes are combined in one acquisition. The same US transducer as in 2D-SWE evaluation was used following the same acquisition protocol as in 2D-SWE measurements. Att.PLUS and SSp.PLUS ROIs were placed in a homogeneous area of the liver parenchyma free of vessels or other structures (Figure 2). Reliable measurements were defined as the median value of 5 measurements, with an IQR/M <30%.

### 2.5. Transient Elastography and Controlled Attenuation Parameter

The FibroScan Compact 530 system was used to perform TE with CAP measurements. The Standard M (3.5 Hz frequency) probe or the XL (2.5 Hz frequency) probe were selected using the automatic probe selection tool software embedded in the device. All patients were evaluated in fasting conditions (for at least 4 h), in supine position, with the right arm in maximum abduction, by scanning the right liver lobe through an intercostal space, in an area free of large vessels or other structures, according to the EFSUMB and WFUMB guidelines [6,7]. Reliable results, representing the median value of 10 valid measurements, with an IQR/M < 30%, are expressed in kPa with values ranging between 2.5 and 75 kPa—for fibrosis and in dB/m, with values ranging between 100 and 400 dB/m—for steatosis [19].

In this study, the cut-off values recommended by Eddowes et al. (2019) in a prospective multicenter study performed in patients with NAFLD, using liver biopsy as gold-standard, were used: F2 ≥ 8.2 kPa; F3 ≥ 9.7 kPa and F4—13.6 [25]. Therefore, the TE cut-off value of 8.2 kPa was considered for the presence of significant fibrosis (F ≥ 2). For steatosis assessment, the cut-off values established by Petroff et al. (2021) were used: S1: 294 dB/m, S2: 310 dB/m, and S3: 331 dB/m, respectively. The cut-off value of 310 dB/m by CAP was considered as indicative for at least significant steatosis (S2–S3) [36].

### 2.6. Statistical Analysis

The statistical analysis was performed using MedCalc Version 19.4 (MedCalc Software Corp., Brunswick, ME, USA) and Microsoft Office Excel 2019 (Microsoft for Windows).

Descriptive statistics were used for demographic, anthropometric, and laboratory findings. The Kolmogorov–Smirnov test was used to establish the distribution of numerical variables. Numerical variables with normal distribution are presented as means ± standard deviation, while variables with non-normal distribution are presented as median values and range. Qualitative variables were presented as percentages and numbers. Parametric tests (*t*-test) were used for the assessment of differences between numerical variables with normal distribution; and nonparametric tests (Mann–Whitney or Kruskal–Wallis tests) for variables with non-normal distribution. The Student’s *t*-test was used to compare the mean LS values by TE and 2D-SWE.PLUS in different fibrosis stages. Chi-square (X2) test (with Yates’ correction for continuity) was used to determine if there are significant differences between the technical success rate of TE and 2D-SWE.PLUS. Linear regression analysis was used to evaluate the correlation between LSMs obtained by TE and 2D-SWE.PLUS and between the values obtained using Att.PLUS, SSp.PLUS, and CAP. Bland-Altman analysis was used to establish the agreement between TE and 2D-SWE PLUS.

Areas under receiver operating characteristic curves (AUROC) were determined for 2D-SWE.PLUS to identify the optimal cut-off points that maximized the Youden index for staging liver fibrosis as compared to TE, which was used as the reference method. The diagnostic performance of SSp.PLUS and Att.PLUS for liver steatosis was estimated using the CAP cut-off value of 310 dB/m for the presence of at least significant liver steatosis (S2–S3), and the optimal cut-off values that maximized the Youden index were determined from the AUROC curve analysis. Rule-out and rule-in cut-off values were also determined from the AUROC curve analysis. Cut-off values that optimized specificity were chosen as rule-in cut-off values, while those that optimized sensitivity were chosen as rule-out cut-off values. Positive predictive value (PPV—defined as the ratio between the true positive cases and all the positive cases), negative predictive value (NPV—defined as the ratio between the true negative cases and all the negative cases) and diagnostic accuracy (defined as the ratio between the sum of true positive and true negative cases and the total number of cases) were determined. 95% confidence intervals (CI) were calculated for each predictive test, and a *p*-value below 0.05 was considered to concede statistical significance.

## 3. Results

### 3.1. Baseline Characteristics

A total of 215 patients with NAFLD were enrolled in the study and underwent multiparametric measurements (mean age: 54.9 ± 11.7, 54.5% were male). In total, 11/215 (5.1%) patients had invalid or unreliable measurements by at least one elastographic technique; therefore, 204 subjects were included in the final analysis. Fibrosis distribution according to TE cut-offs was as follows: 84.4% (172/204) of patients had no or mild fibrosis (F0–1); 3.9% (8/204) of patients had significant (F2) fibrosis; 9.8% (20/204) of patients had advanced (F3) fibrosis and 1.9% (4/204) of patients had liver cirrhosis. The main characteristics of the patients with reliable measurements are presented in Table 1.

### 3.2. Feasibility

Valid LSMs were obtained in 98.6% (212/215) of patients by TE with CAP, in 95.8% (206/215) of patients by 2D-SWE.PLUS/Vi.PLUS and in 98.1% (211/215) of patients by Att.PLUS/SSp.PLUS, respectively. One patient, who had failed LSM with TE, also had failed with 2D-SWE.PLUS, due to the inability to hold their breath. Failure to acquire valid LSMs with 2D-SWE was encountered in 4/11 patients, and it was due to an inhomogeneous filling of the color map (no or little signal). The rest of the unreliable measurements were considered as such because of IQR/M > 30% or of the SI < 90%.

Mean BMI and abdominal circumference were calculated and compared in patients with reliable LSM versus those with unreliable measurements. Both BMI (kg/m^2^) mean values and abdominal circumference mean values were significantly higher for patients with unreliable LSM as compared to those with reliable measurements (35.77 ± 6.43 kg/m^2^ vs. 31.27 ± 5.56 kg/m^2^, *p* = 0.01 and 123.18 ± 4.86 vs. 108.74 ± 11.30, *p* < 0.0001).

Reliable measurements were obtained in 94.9% (204/215) of the included subjects using both TE and 2D-SWE.PLUS. No significant differences between the feasibility of TE and 2D-SWE.PLUS (*p* = 0.14) or between CAP and Att/SSp.PLUS (*p* = 0.98) were found.

### 3.3. Comparison between LSMs Obtained by 2D-SWE.PLUS and TE

The study population was divided into four subgroups according to the fibrosis stage of each subject established based on LS values by TE using the previously mentioned cut-off values, and the mean LS values were calculated for each fibrosis group (Table 2). The mean LS values obtained by TE were significantly higher compared to those obtained using 2D-SWE.PLUS when referring to all the subjects included in the study (overall group), while when comparing the values of LS using the two elastography techniques (TE and 2D-SWE) in each fibrosis group, in the F0–1, F2, and F3 group, the mean LS values were significantly higher for TE compared to 2D-SWE.PLUS, but no differences were found in the F4 group (Table 2).

### 3.4. Correlation between LSMs Obtained by 2D-SWE.PLUS and TE

**Figure 3:**
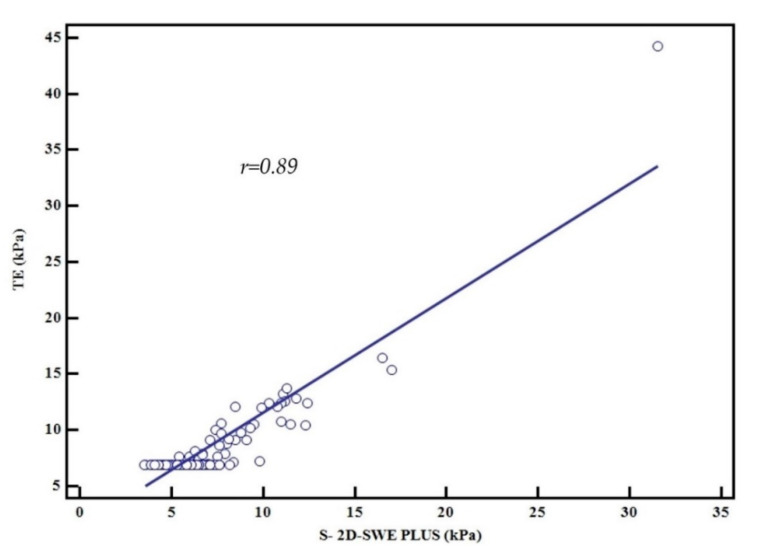
Relationship between the mean values assessed by TE and 2D-SWE.PLUS, Pearson’s correlation coefficient, r = 0.89.

**Figure 4:**
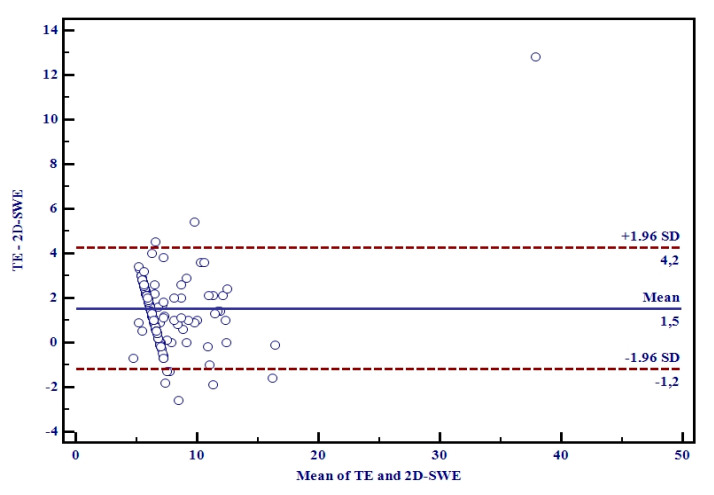
Bland-Altman plots of differences between TE and 2D-SWE.PLUS measurements. The solid line represents the mean of the difference between the elastography measurements taken with the two techniques; the dashed lines define the limit of agreement: the mean difference is 1.5 ± 0.35. The 95% upper and lower LOA were 4.2 and −1.2, respectively.

An excellent correlation was obtained between LSMs by 2D-SWE.PLUS and TE (r = 0.89, R^2^ = 0.79, *p* < 0.0001), meaning that 80% of the TE LSM values can be explained by 2D-SWE.PLUS LSM values (Figure 3). The Bland–Altman analysis showed that the mean difference in LSMs between 2D-SWE.PLUS and TE was 1.5 ± 0.35 kPa. The 95% upper and lower limits of agreement (LOA) were 4.2 and −1.2 kPa, respectively (Figure 4).

### 3.5. Diagnostic Performance of 2D-SWE.PLUS for Liver Fibrosis Staging Using TE as Reference

**Figure 5:**
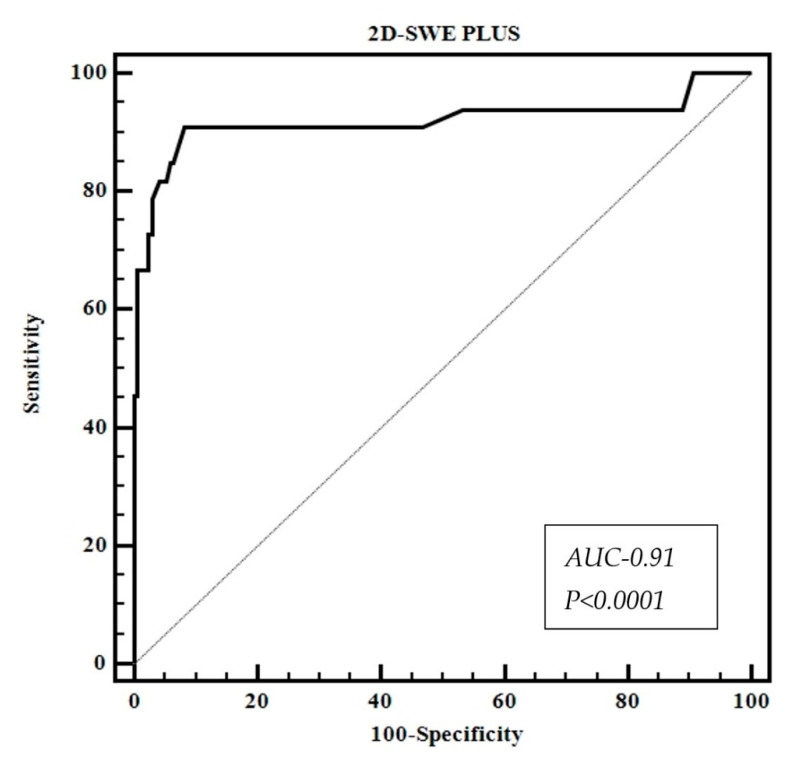
Performance of 2D-SWE.PLUS for predicting significant fibrosis (F ≥ 2).

2D-SWE.PLUS showed great performance (AUC-0.91) for the diagnosis of significant fibrosis (F ≥ 2) (Table 3, Figure 5). The optimal LSM cut-off value determined by the Youden Index and their corresponding sensitivity, specificity, negative predictive values, and positive predictive values of 2D-SWE.PLUS in identifying significant fibrosis are illustrated in Table 3.

### 3.6. Performance of Att.PLUS and SSp.PLUS for Predicting the Presence of Liver Steatosis Using CAP as Reference

SSp.PLUS correlated better than Att.PLUS with CAP values: (r = −0.74, *p* < 0.001) vs. (r = 0.45, *p* < 0.001). The optimal Att.PLUS and SSp cut-off values for predicting S2-S3 steatosis and their corresponding sensitivity, specificity, negative predictive values, and positive predictive values are summarized in Table 3.

The “ruling in” SSp.PLUS cut-off value (which optimized specificity) (SSp ≤1516 m/s) showed 98.36% specificity, with 58.74% sensitivity, for predicting the presence of at least significant steatosis (S2–S3).

The “ruling out” of SSp.PLUS cut-off value (which optimized sensitivity) (≥ 1559 m/s) showed 95.10% sensitivity, with 32.8% specificity, to exclude the presence of at least significant steatosis (S2–S3).

Univariate and multivariate statistical analyses were used to examine the relationships between the SSp.PLUS values and the following parameters: BMI, abdominal circumference, AST, ALT, Att.PLUS values, cholesterol levels, triglycerides levels, and CAP values. Univariate analysis showed that BMI (*p* = 0.002), abdominal circumference (*p* < 0.001), CAP values (*p* < 0.001), and Att.PLUS values (*p* < 0.001) were independently associated with SSp.PLUS values.

In multivariate regression analysis, only abdominal circumference (*p* < 0.001) and CAP values (*p* < 0.001) were independently associated with SSp.PLUS values.

To analyze whether there is a difference between the mean values of SSp.PLUS in patients with diabetes mellitus or with arterial hypertension compared to those without, the study group was divided into two groups based on the presence of diabetes mellitus or arterial hypertension, and the mean SSp.PLUS values were calculated in both groups. Mean values were significantly lower in subjects with diabetes mellitus (n = 56), compared to those without (1510.3 ± 25.1 m/s vs. 1529.7 ± 28.4 m/s, *p* < 0.0001). However, no significant differences were found between SSp.PLUS mean values in subjects with arterial hypertension (n = 128) compared to those without (1522.9 ± 28.5 m/s vs. 1526.7 ± 29.3 m/s, *p* = 0.358).

The mean CAP values were also compared based on the presence of diabetes mellitus or arterial hypertension. CAP mean values were significantly higher in the group of subjects with diabetes mellitus compared to those without (346.9 ± 40.4 dB/m vs. 321.6 ± 42.8 dB/m, *p* = 0.0002), while no significant differences were found between CAP mean values in the group of subjects with arterial hypertension compared to those without (330.8 ± 45.6 dB/m vs. 324.7 ± 39.9 dB/m, *p* = 0.327).

### 3.7. Performance of Vi.PLUS. Influence of Patients’ Characteristics on Vi.PLUS

**Figure 6:**
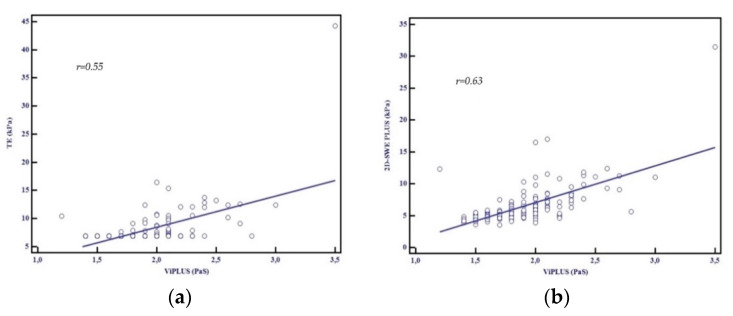
(**a**) Relationship between the mean values assessed by TE and Vi.PLUS, Pearson’s correlation coefficient, r = 0.55; (**b**) Relationship between the mean values assessed by 2D-SWE.PLUS and Vi.PLUS, Pearson’s correlation coefficient, r = 0.63.

A good correlation between Vi.PLUS measurements and LSM by TE (r = 0.55, *p* < 0.001) and by 2D-SWE.SSI (r = 0.63, *p* < 0.001) was found (Figure 6a,b).

Univariate and multivariate statistical analyses were used to examine the relationships between the Vi.PLUS values and the following parameters: BMI, abdominal circumference, AST, ALT, and LSM by TE, and LSM by 2D-SWE.PLUS. The univariate regression analysis showed an independent association between Vi.PLUS measurements and the following parameters: LSM by 2D-SWE.PLUS (*p* < 0.001); BMI (*p* < 0.001); abdominal circumference (*p* < 0.001); and LSM by TE (*p* < 0.001). AST (*p* = 0.62) and ALT (*p* = 0.49) were not associated with Vi.PLUS measurements. In multivariate regression analysis, BMI (*p* < 0.0001) and LSM by 2D-SWE.PLUS (*p* < 0.0001) were independently associated with Vi.PLUS values.

## 4. Discussions

With an estimated global prevalence of 25% and increasing, NAFLD became an important economic and social burden. The development of liver fibrosis was found to be the most important factor in predicting clinical decompensation as well as the development of complications such as hepatocellular carcinoma and death. Thus, quantitative assessment of liver fibrosis and steatosis content using non-invasive techniques is of great importance in evaluating and staging NAFLD [1].

ShearWave PLUS is a new elastography software embedded in the Aixplorer Mach 30 ultrasound system, which includes a new quality criterion, the SI (Stability Index tool). In a study published by Hong et al. (2018), increased reliability and reduced measurement variability were obtained when the SI was used to acquire LMS in healthy children [37]. To our knowledge, to date, from the studies published that have evaluated the performance of 2D-SWE by SSI in a cohort of NAFLD patients, no study used this new quality criterion.

Using the old 2D-SWE method developed by SSI, reliable LSM can be achieved in 90–98% of patients [16,17,18]. A study published by Cassinotto et al. (2016) that included only NAFLD patients showed that valid LSM measurements could be obtained in 87% of patients [38]. The present study shows that measuring liver fibrosis using the new 2D-SWE.PLUS embedded into SSI’s Aixplorer Mach 30 is a highly feasible method with a 95.8% feasibility. There were no significant differences between TE and 2D-SWE PLUS’s technical success rate, our results being consistent with previously published data [16,17,18,39].

A prospective comparative study between four SWE techniques revealed that values obtained using 2D-SWE by SSI correlated significantly with TE values (r = 0.86, *p* < 0.0001) [40]. Another study that enrolled 349 consecutive patients with chronic liver diseases who underwent liver biopsy and 2D SWE from SSI showed that 2D-SWE correlated significantly with histological fibrosis score (r = 0.79, *p* < 0.00001). A prospective study published by Iijima et al. (2019) that included 119 consecutive patients with chronic liver disease showed that AUROCs for predicting significant fibrosis (≥F2) and cirrhosis (F4) based on SWE embedded in five different ultrasound devices using TE as the control method were over 0.8 and 0.9, respectively. Additionally, the correlation coefficients obtained between TE values and SWE values were all over 0.8 [41]. In the current study, the linear regression analysis showed an excellent correlation between LSMs obtained by 2D-SWE PLUS and TE (r = 0.89, R^2^ = 0.79, *p* < 0.0001).

A comparative study that included 291 patients with NAFLD, who underwent LSM using *p*-SWE from Siemens, 2D-SWE from SSI and TE as compared to liver biopsy was published by Cassinotto et al. (2016) [38]. The AUROC for 2D SWE from SSI was 0.89 for diagnosing significant fibrosis (F ≥ 2) with similar cut-off values to TE for predicting significant fibrosis: 6.3 vs. 6.2 kPa. Herrmann et al. (2018) published a meta-analysis based on individual patients’ data, which evaluated 2D-SWE (SSI) using liver biopsy as reference; 2D-SWE (SSI) showed a good performance in fibrosis assessment in NAFLD patients with an AUROC of 0.85 for diagnosing significant fibrosis (F ≥ 2) and of 0.95 for diagnosing cirrhosis [20]. The best cut-off for diagnosing F ≥ 2 was 7.1 kPa. Similar results were presented in other three meta-analyses [42,43,44]. In this study, the calculated 2D-SWE.PLUS cut-off value for significant fibrosis (F ≥ 2), using TE as the reference method, was 7 kPa.

Several quantitative ultrasound-based methods are available for hepatic steatosis evaluation. The first method used was CAP. Numerous studies that used liver biopsy or magnetic resonance imaging-derived proton density fat fraction (MRI-PDFF) as the reference method showed a good performance of CAP for liver steatosis assessment [23,24,27,45,46].

New software programs, embedded in different ultrasound devices, measuring the attenuation of ultrasound beams using B-mode guidance were developed. Several methods were analyzed: Attenuation (ATT) by Hitachi Ltd. (Tokyo, Japan), ultrasound-guided attenuation parameter (UGAP) by GE Healthcare (Wauwatosa, USA), and Attenuation imaging (ATI) by Canon Medical Systems (Tochigi, Japan) [47,48,49,50]. Good correlations were found between ATI, UGAP, and the control techniques used (r = 0.75, respectively r = 0.81). However, ATT presented a moderate correlation of r = 0.50 [47,48,49,50]. In our study, Att.PLUS—the technique that quantifies the sound beam attenuation—moderately correlated the control method: r = 0.45 (*p* < 0.001), similar to ATTs’ (Hitachi) performance.

Ex vivo studies have revealed that the longitudinal speed of sound is lower in fat than in the healthy liver parenchyma [51]. Hepatic steatosis was associated with a decrease in the intrahepatic speed of sound [52]. An ultrasound-based technique that measures the speed of sound in the liver has been evaluated in a proof of concept study [53]. This method’s ability (Aixplorer ultrasound system) to assess liver steatosis was analyzed using magnetic MRI-PDFF as the control method [54]. A cut-off value ≤ 1.537 mm/µs had 80% sensitivity and 85.7% specificity in identifying steatosis. The speed of sound showed a solid correspondence to MRI-PDFF (R^2^ = 0.73) [54].

In our study, a strong correlation between CAP and SSp.PLUS values was observed (r = −0.74, *p* < 0.001). The best SSp cut-off value determined by the Youden Index for predicting the presence of significant hepatic steatosis (S ≥ 2) using the CAP cut-off value of 310 db/m was 1524 m/s, with an AUROC of 0.88, sensitivity (Se) = 75.5%, specificity (Sp) = 93.4%. The univariate analysis showed that BMI, abdominal circumference, CAP values, and Att.PLUS values were independently associated with SSp.PLUS. In multivariate regression analysis, the model including abdominal circumference and CAP values was associated with SSp.PLUS values. SSp.PLUS mean values were significantly lower in subjects with diabetes mellitus (n = 56) compared to those without (1510.3 ± 25.1 vs. 1529.6 ± 28.4 m/s, *p* < 0.0001). Although more studies are needed, our data suggest that this technique can be used in clinical practice to assess hepatic steatosis.

Inflammation plays an essential role in the development of liver fibrosis [32]. Tissue viscosity has been non-invasively evaluated in only a small number of studies. Deffieux et al. (2015) first published a study on liver viscosity using an US imaging system (Aixplorer; Supersonic Imagine, Aix-en-Provence, France) [55]. The results showed that viscosity had less predictive value in staging fibrosis than liver stiffness and was a modest predictor of disease activity and steatosis levels. The study published by Chen et al. (2013) on liver viscosity evaluated both elasticity (kPa) and viscosity (Pa·s) with an ultrasound system (iU22, Philips Healthcare, Andover, MA, USA) in patients with various chronic liver diseases. The results showed that viscosity had less predictive value in staging fibrosis than elasticity [56]. In a study published by Sugimoto et al. (2018), in the multivariate analysis performed with histologic features as independent variables, the fibrosis stage was found to be significantly related to SW speed (*p* = 0.037), and lobular inflammation grade was significantly related to dispersion slope (*p* = 0.022). Elasticity was found to be more useful than viscosity for predicting the stage of fibrosis, and viscosity was found to be more useful for predicting the degree of necroinflammation [57].

In our study, the univariate regression analysis showed an independent association between Vi.PLUS measurements and LSM by 2D-SWE.PLUS, BMI, abdominal circumference, and LSM by TE. AST and ALT were not independently associated with Vi.PLUS measurements. In multivariate regression analysis, BMI and LSM by 2D-SWE.PLUS were associated with Vi.PLUS values.

A limitation of this study is the low prevalence of advanced stages of fibrosis (F3–F4); for this reason, cut-off values could not be calculated for all stages of fibrosis. Our study included “a real-life cohort” of consecutive NAFLD patients with a high prevalence of mild and no liver fibrosis (F0–F1). Moreover, a high AUROC value for predicting F ≥ 2 was obtained in our study; this result could have been biased by the unequal distribution of liver fibrosis in our cohort. In line with these limitations, Chan et al. demonstrated in a retrospective analysis that sensitivity and negative prediction value decrease as the percentage of the target population increases [58]. Another limitation of this study was that liver biopsy was not available. However, the study was meant to evaluate a new ultrasound-based technique compared to a validated method for fibrosis and steatosis quantification. In addition, while using the published cut-off values for detecting different grades of liver steatosis recommended by Petroff et al. (2021), 20.7% (40/193) of the subjects included in our study were classified as S0 (no steatosis), although the presence of liver steatosis detected by abdominal ultrasound was used as inclusion criteria for NAFLD [36]. This discrepancy could be explained by the fact that the cut-off value used in our study for detecting S1 (294 dB/m) is much higher than the previously recommended cut-off of 248 dB and has better performance for ruling-in mild liver steatosis S1 (low NPV, high PPV) [23,36].

In practice, in this moment, ultrasound became a non-invasive multiparametric tool that allows assessment of the hepatic structure, the evaluation of liver fibrosis, the detection and quantification of hepatic steatosis and, finally, the evaluation of viscoelastic properties. In NAFLD patients, MPUS is of real value for stratifying subjects, looking to the severity of the disease.

## 5. Conclusions

MPUS is a highly feasible method that allows complex a evaluation of liver fibrosis, steatosis, and viscosity, in a short time, in NAFLD patients. 2D-SWE.PLUS strongly correlates to TE, having an optimal cut-off value for predicting F ≥ 2 of 7 kPa, while SSp.PLUS correlated better than Att.PLUS with CAP. Further studies are needed to confirm our results, using liver biopsy as control when possible.

## Figures and Tables

**Table 1:** Characteristics of subjects with reliable LSMs by both methods.

Parameter (Mean ± SD)	n = 204
Age (years)	54.7 ± 11.7
Gender	
Males	111/204 (54.4%)
Females	93/204 (45.6%)
BMI (kg/m^2^)	31.2± 5.5
Abdominal Circumference (cm)	108.7 ± 11.3
AST (UI/L)	39 ± 27.7
ALT (UI/L)	51.1 ± 41.1
GGT (mg/dL)	89.4 ± 97.1
Cholesterol (mg/dL)	201.4 ± 49.1
LDL_c_ (mg/dL)	126.8 ± 45.9
HDL_c_ (mg/dL)	43.9 ± 10.7
Triglyceride (mg/dL)	198.5 ± 155.1
CAP (dB/m)	328.5 ± 43.6
LS by TE (kPa)	7.7 ± 3.1
2D-SWE.PLUS (kPa)	6.2 ± 2.7
SSp.PLUS (m/s)	1524.3 ± 28.8
Att.PLUS (dB/cm/MHz)	0.4 ± 0.1
Vi.PLUS (Pa.S)	1.8 ± 0.3
**Liver fibrosis distribution by TE**	
F0–1	84.4% (172/204)
F2	3.9% (8/204)
F3	9.8% (20/204)
F4	1.9% (4/204)
**Liver steatosis distribution by CAP**	
S0	20.6% (42/204)
S1	9.3% (19/204)
S2	21.1% (43/204)
S3	49.0% (100/204)

Data are presented as number and percentage or mean ± standard deviation; ALT = alanine aminotransferase, AST = aspartate aminotransferase, BMI = body mass index, LS = liver stiffness, TE = Transient Elastography, CAP = Controlled Attenuation Parameter, 2D-SWE.PLUS = Two-Dimensional Shear Wave PLUS Elastography by SuperSonic Imagine, SSp.PLUS = Sound Speed Plane-wave UltraSound, Att.PLUS = Attenuation Plane-wave UltraSound, Vi.PLUS = Viscosity Plane-wave UltraSound.

**Table 2:** Differences between mean LSMs by TE and 2D-SWE.PLUS in different fibrosis stages.

Fibrosis Stage	TE (kPa)	2D-SWE.PLUS (kPa)	*p* Value
Overall	7.76 ± 3.10	6.23 ± 2.73	*p* < 0.0001
F0–1	6.88 ± 0.61	5.48 ± 1.04	*p* < 0.0001
F2	8.97 ± 0.21	7.25 ± 1.64	*p* = 0.0107
F3	11.47 ± 1.15	9.90 ± 1.67	*p* = 0.0013
F4	22.45 ± 14.60	19.07 ± 8.67	*p* = 0.70

TE = Transient Elastography; 2D-SWE.PLUS = Two-Dimensional ShearWave PLUS.

**Table 3:** Performance of 2D-SWE.PLUS for predicting significant fibrosis (F ≥ 2) and the performance of Att.PLUS and SSp.PLUS for predicting S2–S3 steatosis.

2D-SWE.PLUS for Predicting Significant Fibrosis (≥F2)									
	**Cut-off Value**	**AUC** **(95%CI)**	**Se (%)**	**Sp** **(%)**	**PPV** **(%)**	**NPV** **(%)**	**LR+**	**LR−**	***p***
≥F2	7 kPa	0.91 (0.87–0.95)	90.9	91.8	68.2	98.1	11.1	0.09	*p* < 0.0001
**Att.PLUS and SSp.PLUS for predicting S2-S3 steatosis**									
Att.PLUS	0.5 (dB/cm/MHz)	0.72 (0.66–0.78)	53.1	82.0	87.4	42.7	2.95	0.57	*p* < 0.0001
SSp.PLUS	<1524 m/s	0.88 (0.82–0.92)	75.5	93.4	96.4	62.0	11.5	0.26	*p* < 0.0001

AUROC = area under the receiver operating characteristics curve, CI = confidence interval, Se = sensitivity, Sp = specificity, PPV= positive predictive value, NPV = negative predictive value. 2D-SWE.PLUS = Two-Dimensional ShearWave PLUS Elastography, SSp.PLUS = Sound Speed Plane-wave Ultrasound, Att.PLUS = Attenuation Plane-wave Ultrasound.

## Data Availability

The data underlying the findings of the study are available on request to the corresponding author (e-mail address: bende.felix@umft.com).
